# An Inventory of Deceased Donor Family Care and Contact Between Donor Families and Recipients in 15 European Countries

**DOI:** 10.3389/ti.2021.10188

**Published:** 2022-01-10

**Authors:** Tineke Wind, Nichon Jansen, Anne Flodén, Bernadette Haase-Kromwijk, David Shaw, Dale Gardiner

**Affiliations:** ^1^ Maastricht University Medical Centre, Maastricht, Netherlands; ^2^ Institute of Health and Care Science, Dutch Transplant Foundation, Leiden, Netherlands; ^3^ Institute of Health and Care Science, University of Gothenburg, Gothenburg, Sweden; ^4^ Department of Anaestesiology, Södra Älvsborgs Hospital, Borås, Sweden; ^5^ Institute of Biomedical Ethics, University of Basel, Basel, Switzerland; ^6^ Department of Health, Ethics and Society, Care and Public Health Research Institute, Maastricht University, Maastricht, Netherlands; ^7^ Nottingham University Hospitals NHS Trust, Nottingham, United Kingdom

**Keywords:** organ donation, donor family care, contact donor and recipient, remembrance ceremonies, family after care

## Abstract

Families of organ donors play an important role in the deceased organ donation process. The aim of this study was to gain insight into donor family care by creating an inventory of practice in various European countries. A questionnaire about donor family care and contact between donor families and recipients was developed. Representatives of the organ donor professionals of 15 European countries responded (94%). The donor coordinator plays a key role in care for the donor family. All countries provide information about the donation results to the families, although diminished due to privacy laws. Anonymous written contact between donor families and recipients is possible in almost all countries and direct contact in only a few. Remembrance ceremonies exist in most countries. Half of the respondents thought the aftercare could improve. This first inventory shows that differences exist between countries, depending on the organisation of the donation process, the law and the different role of the professionals. Direct contact between donor families and recipients is rarely supported by the donation organisation. To date there has been limited research about the experience of donor family aftercare and we would urge all donation organisations to consider this as a priority area.

## Graphical Abstract

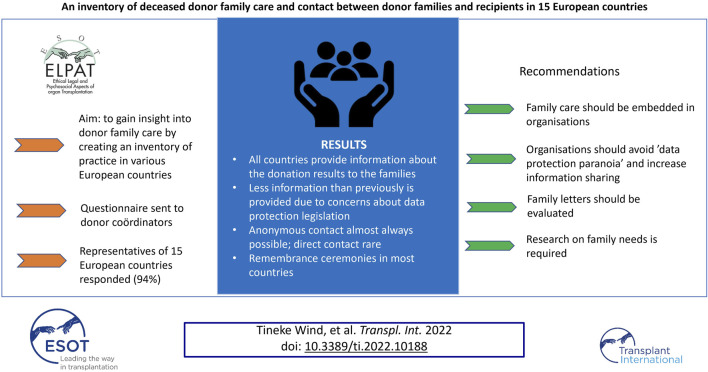

## Introduction

Organ transplantation is a well-accepted medical treatment for organ failure. The main source of donor organs is patients dying in the intensive care unit (ICU), after declaration of brain death (donation after brain death, DBD), or after withdrawal of life sustaining treatment (donation after circulatory death, DCD). In the ICU, “patient-centred care” is the predominant model, which means that individual’s specific health needs and desired health outcomes are the driving force behind all health care decisions and quality measurements (1). Patient-centred care includes family-centred care, as most ICU patients cannot make decisions or communicate for themselves due to the severity of the illness and sedation (2). Families in this context are not limited to blood relations or a singular unit but are composed of various individuals who are close to the deceased.

If the decision is taken that ICU treatment is futile, the end-of life care process will start. Organ donation is an integral part of end-of life care for many patients (3). The family of a potential organ donor plays an important role in the deceased organ donation process. Depending on the consent system of the country in question, information regarding the wish of the patient or consent may be needed from the family (4, 5) and in most countries the agreement or support of the family is sought before donation proceeds. Communication with the family is therefore important to gain insight in the decision or attitude of the patient regarding organ donation (6, 7).

Organ donation can be overwhelming and stressful for families for many reasons, including the duration of the process and grief at the death of a loved one (8). However, donation can also lead to longer term positive outcomes, particularly as the knowledge that the donation helped other people can lessen the burden of bereavement (9). This highlights the importance of communication with, and care of, the donor family during and after the donation process (9, 10).

The aim of this study is to gain insight into donor family care by creating an inventory of practice in various European countries. We focus on two aspects: the formal communication with the family during and after the donation process and the possibility of contact between donor families and recipients.

## Methods

The Deceased Donation Working Group of ELPAT (the Ethical, Legal, and Psychological Aspects of Transplantation section of the European Society for Organ Transplantation) developed a questionnaire. The items of the questionnaire were based on our professional experiences with the donation process. The questionnaire contained two parts: communication and care for donor families during and after the donation and contact between donor families and recipients.

The first part of the questionnaire contained 16 questions, of which 13 were multiple-choice questions with the ability to provide additional comments, and three open questions. Subjects covered included: guidance of the family during the donation process, information and care provided to families after donation and the provision of remembrance ceremonies for the family.

The second part contained eight questions, of which six were multiple-choice and two open questions. Subjects covered included: contact, what kind of contact, possibility and experience of meetings between donor families and recipients.

Pilot testing of the questionnaire was done with five organ donor coordinators, and using their comments, minor modifications were made to the questionnaire. For each country, one representative was approached by mail or telephone during early 2019; the study was explained and consent was obtained to participate. Each representative was chosen because of their anticipated in-depth knowledge of their country’s donation process, and their ability to obtain information from a diverse group to reduce heterogeneity and subjectivity in the replies.

If there were, according to the representative likely to be regional differences in the country, additional representatives were approached as required.

## Results

From the 16 approached countries, 15 responded (94%). In three countries (Belgium, France and Netherlands), more questionnaires were returned from different regions, to explore regional differences. Respondents were all experts on the donation process in their country, although their specific job titles varied; for example, organ donor coordinators, transplant coordinators or specialist nurse for organ donation. As these names differ for the person who fulfills the similar task of coordinating the donation process, we use for clarity in this article one term for all the above job titles: donor coordinator (DC).

### DC Contact With Family in Relation to Donation Consent

The organisation of the donation and the roles of the professionals involved in the donation process differ by country. Communication with and support of the donor family in the ICU was, in most countries, organised in cooperation between the DC, the intensivist and the ICU nurse. The DC coordinates the donation process on the ICU, has contact with the family and coordinates the organ retrieval procedure.

Table 1 outlines the timing of DC contact with the family in relation to consent for donation. In five countries there is no direct contact between the DC and the family (Denmark, Hungary, Sweden, Norway, and some regions of Belgium) and the DC coordinates the donation process at distance from an office, in close contact with the intensivist. In three countries (Croatia, Germany and Netherlands) only after consent is given for donation can there be contact between the DC and the family. In eight countries, contact between the family and the DC is possible before there is consent for donation. For example, in the U.K. the DC (called the specialist nurse—organ donation) plays an important and active role in the request for donation. Support and guidance of the family on the ICU during the donation process is provided by the intensivist and ICU nurse in all countries, while in 11 countries also the DC is involved in family guidance throughout the donor procedure.

**TABLE 1:** Countries and first contact by donor coordinator with family, remembrance ceremonies and meeting transplant recipient.

Country	DC contact with family in relation to donation consent	Remembrance ceremonies organised	Meeting donor family and recipient
Belgium	Before/after/no contact*	Yes	Yes
Croatia	After	No	No
Denmark	No contact	Yes	No
France	Before	Yes	No
Finland	Before	No	No
Germany	After	Yes	No
Hungary	No contact	Yes	No
Iceland	Before	Yes	No
Netherlands	After	Yes	Yes
Norway	No contact	Yes	No
Slovenia	Before/after	No	No
Spain	Before/after	Yes	No
Sweden	No contact	No	Yes
Switzerland	Before/after	Yes	No
United Kingdom	Before	Yes	Yes

*Regional differences.

### Care for Donor Families After Donation

All countries provide information to the donor family after the donation procedure. This information is given by letter, by telephone and in some countries face to face at the family home or in the hospital. The information is provided by the DC; only in the four countries where there is no contact between the DC and the family, the intensivist provides the information.

Respondents from most countries say the way in which information is provided depends on family wishes. In three countries, a national organisation provides the information (Hungary, Slovenia, United Kingdom). In some countries, the donor family is invited to the hospital a few months after the donation procedure, to evaluate and discuss the donation process.

The kind of information that is provided after the donation procedure varies; information about which organs/tissues are transplanted, information about the recipients gender, age or health. In two countries (Hungary and Finland), only a standard “thank you letter” is sent without any information about the recipients. During the last years, the information provided has reduced in many countries (n = 7), e.g., now the information about age, gender, time on the waiting list, and the health of the recipient, is limited. Some countries now only provide the information that the organ is transplanted or not. A reason suggested by participants for less information sharing was data protection legislation. Some stated that this is a pity, because less meaningful letters are sent to the family. Another factor suggested was social media, due to the fear that donor families will search for recipients’ information. This was mentioned for two countries.

The representatives from six countries expressed overall satisfaction with the care provided for donor families (Netherlands, Belgium, United Kingdom, France, Finland Iceland). The main concern from the other country representatives who were not satisfied was that there was no or not enough aftercare for donor families. Structural organised care for families after donation was missing, were some comments, like: “There is no organised care, donor family care should be implemented.” “More national follow up of donor families is needed.” A lack of structured organised after care was a frequent observation, highlighting there was too much variation per hospital or region. Comments: “There should be more follow up with donor families after they leave the hospital, to learn more about their grieving process and implement the lessons learned.” Information was lacking concerning the effects, positive or negative, of the donation to this grieving process. In some countries the opinion was that donor after care is too variable per region or hospital and should be implemented on a national level, to guarantee donor after care for all donor families. Comments: “smaller hospitals are less experienced; there is a wish to give more support to all families, not only to those who ask for help or information.”

We were interested if there was a difference in care for donor families on the ICU and families of regular ICU patients who die in the ICU. From the respondent countries, almost half are of the opinion that the care differs. The general opinion was that more attention is given to donor families during the donation process, especially if there is a DC present to coordinate the donor procedure, or a trained professional to give information and support to the family and who can help them in the beginning of the grieving process. Donor families also receive more care after the donation (aftercare) than families of deceased ICU patients, was the opinion of seven countries. “Donor families are invites to special ceremonies,” “donor families receive ‘thank you letters.’” “Yes, the care is different, donor families are invited to the donor hospital 6 weeks after the donation, to talk about their experience.” Families from regular ICU patients who die don’t have these special ceremonies in general. However, some state the care is equal, also families of non-donors are invited to the hospital for after care.

### Remembrance Ceremonies for Donor Families

In 11 countries remembrance ceremonies are organised for donor families, only in four countries (Croatia, Finland, Slovenia, Sweden) was this not the case (Table 1). The ceremonies are organised at different levels: at a national level, a regional level, or at a hospital level. Examples of these different ceremonies are: a donor family day, a transplant honours day, a donor memorial day, a remembrance walk with donor families, a national donor monument, a hospital donor monument, a donor tribute evening. Sometimes smaller ceremonies are organised at a hospital level, like a farewell ceremony organised by a priest. There are hospitals that have a monument in their hospital in honour of their donors. In one country (the United Kingdom) a posthumous national award consisting of a special certificate and pin is offered to all the donor families, to pay respect to the donor through the donor family. During the annual ceremonies in some countries, recipients are also present as well as representatives from transplant centres to give support to families.

Who is responsible for organising the ceremonies differs per country: private organisations, a recipient organisation, local organisations from donor hospitals, or the event can be organised by the transplant foundation, or regional teams. All countries who provide ceremonies, state that they were satisfied with these ceremonies. Comments on the ceremonies: “very helpful for donor families,” “families are happy with the attention,” “well visited meetings,” “important to share experiences and emotions with other families.” According to some countries improvement could be the presence of a professional during the meetings, like a DC, to answer questions and give specific information.

### Contact Between the Donor Family and Recipient(s)

In all countries but one (Croatia), written anonymous contact between recipient and donor family is possible, through an intermediate, the DC. In one country (Switzerland) a website was developed, where recipients and donor families can post their thoughts, thanks, experiences, and histories. Here was also a guide/template for a “thank you letter” to be used for transplant patients to a donor family. Initiative for contact is taken more by recipients than by donor families.

Four countries responded that a formalised process exists for donor family and recipient(s) to meet face-to-face (Table 1). However, the circumstances and conditions differ. For example, in the Netherlands, this is only possible through an organisation where donor families and recipients can report themselves, without involvement of professionals. Because anonymity is regulated by law, professionals cannot be involved. The organisation matches the donor family and recipient and arrange the meeting. In Belgium during donor day, a meeting is possible. In Sweden meetings happen, but without health care personnel. In the United Kingdom, the meetings are held in a mutual convenient place, well prepared with the support of the DC. Because the satisfaction of the donor family is not routinely measured, most countries state that family experience are not known. Two countries (the United Kingdom and Belgium) are positive about the meetings. Comments of other countries suggests that there is a lot of discussion about meetings between donor families and recipients. Comments included:

“Is this a good thing”?

“Should there be a role for the health care professionals during these meetings?” “Expectations should be well managed”

“Is the motivation of the donor family and the recipient the same?”

“Meetings don’t feel right, not intent to cooperate as a professional.”

“What if there is some pressure felt from the donor family to the recipient”?

“Meetings can only be possible if the donor family and recipient find each other through social media.”

A few general comments were made, and suggestions to improve the care for donor families. For example, offering the family a conversation with a psychologist, if they are in need for support or to be more active in approaching recipients to send a kind of thank you letter to the donor family.

### Letters to Donor Families

To be able to compare if the information provided to the donor families from the DC changed over the years, we asked participants to send examples of two letters: a recent letter and one from approximately 10 years ago. Letters were received from five countries. In four countries, the letters were changed, and in all of these cases less information was given about the recipients. Information about time on the waiting list, age (only an age period) or health information about the recipient was no longer given in the latest letters. The recent letters were simpler and more straight forward, consisting only of information concerning whether the transplantation of the organ was successful, with no more detailed information about the health process of the recipient. The reason for this change was reported as being stricter privacy legislation in the different countries.

## Discussion

This study is the first survey about the care of donor families, during and after the donation process and contact between the donor families and the recipients in 15 European countries. It shows that there is a variability between countries, and in some countries small differences between centres or regions. For example, the role of the DC and the moment this professional is participating in the donation process. Depending on the way donation is organised there are also differences in the communication with and care of the donor family. Communication during the donation process with the family is mostly led by the DC; only in a small number of countries is there no direct contact between the DC and the donor family. Generally, DC contact with the donor family starts after consent for donation, though a minority of DCs have contact before consent. There is some evidence that early contact with the family and involvement of the DC in the request for donation can have a positive effect on the perceived support for the family and the consent rate for donation (11).

The amount of information provided to the family about the outcome of the donation and transplantations depends on the legislation and its interpretation, in a specific country. In several countries this information has become more limited in recent years due to data protection legislation. This means that less information about the recipients can be given, mainly restricted to age range, transplant outcomes and sometimes gender of the recipients. It is possible that concerns about privacy and strict interpretations of data protection legislation may not be justified; if a recipient consents to the processing of more data in order to facilitate higher levels of communication with donor families, this would be permitted by the General Data Protection Regulation. However, different countries may have stricter national laws in place, or transplant professionals may be being given legal advice that takes a very strict interpretation of data minimisation where that is not necessary.

Face-to-face meetings between donor family and recipients only take place in a few countries, but there is concern about the impact these meetings can have on both donor families and recipients. There are some studies about the contact between the donor families and transplant recipients (12, 13). Outcomes of the studies differ, but benefits are seen from contact; letters from recipients to donor families are appreciated by donor families and contribute to positive feelings. Expressing gratitude to the family of the person who made the donation possible can be important. However, there are also several reasons for not having contact: protection from emotional stress, not to be reminded of this stressful period and the loss of the loved one, and the wish to leave a difficult period behind. Families who met with recipients, reported that it eased their pain and gave some positive meaning to their loss (14). On the other hand, negative feelings, such as disappointment in the person who received the organ, can also occur. In most countries surveyed, anonymity must be assured and the law is perceived as preventing healthcare professionals from facilitating direct contact between donor families and recipients.

The aftercare given to donor families can be different from the care given to non-donor families on the ICU. This could be influenced by providing more and longer intensive contact with the donor family, more extensive conversations and explanations about the donor procedure by the intensive care staff and coordinator of the donation procedure. In the literature, families who consented to donation felt more supported than families of non-donors (15). Increased satisfaction of families of ICU patients is seen in previous studies, when a family support coordinator is brought in, an extra person who cares for the needs of the family (16). The positive effect of such a person is also seen in the consent rate for donation (17). In this study, almost half of the respondents still thought there was need for improvement of the aftercare for donor families. Too little is known about the impact of the donation procedure on the grieving process. Studies that focus on the impact of organ donation on the grieving process, also show that the act of donation can assist families in their grief (18).

Remembrance ceremonies exist for donor families in almost all countries, and the impression of the DC is that the ceremonies benefit the donor family, but satisfaction with the ceremonies is not routinely measured. There is attention to the needs of donor families, but a lack of specific information about these needs is also mentioned. No specific studies are performed to measure the impact of ceremonies on the donor family.

Data collection was completed before the COVID pandemic, so it had no impact on the results. However, the pandemic may have affected the provision of donor family support, such as public remembrance ceremonies, which are temporarily reduced.

### Reflections and Recommendations

Given the results of our research, we can make several recommendations. Donor family aftercare is an essential part of the donor process and should be delivered in a structured way and embedded into the organisational process. In order to establish best practice for a country, research on family views is needed. What services do donor families need? This research can be done using groups, such as donor family advisory group, or interviews with donor families. Research should also be conducted to evaluate letters from a donor family perspective; what information benefits the donor family? Are meetings between recipients and donor families beneficial for both? Those working with donor families and recipients should reflect on this inventory, and comparative practices, and consider whether they are meeting the needs of donor families. Furthermore, organisations can tend toward risk aversion in data protection at the expense of helping families; this tendency should be avoided. Research evaluating the impact of COVID on donor family care should also be performed.

### Conclusion

This first inventory of 15 European countries about the care provided to donor families during and after donation and contact between donor families and recipients, shows, as expected, that there are differences between the countries. These differences depend on the organisation of donation, the law (and its interpretation) and the different roles of the professionals involved in donation. Donor family aftercare is provided in all countries and some countries provide remembrance ceremonies. In most countries, direct contact between donor families and recipients is not supported by the donation organisation. To date there has been limited research about the experiences and satisfaction of donor family aftercare and we would urge all donation organisations to consider this as a priority area.

### Strengths and Limitations of the Study

This is the first multi-country study to compare the way care to donor family and aftercare is provided to donor families and contact between donor families and recipients, which provides valuable insights. Since this is a first inventory, it provides only an initial overview of the different aspects. More research is necessary to explore in depth in how communication and aftercare is given, and the experiences and satisfaction of the donor families, with including possible suggestions for improvement.

### Participating Countries

Belgium, Croatia, Denmark, Finland France, Germany, Hungary, Iceland, the Netherlands, Norway, Slovenia, Spain, Sweden, Switzerland, United Kingdom.

## Data Availability

The raw data supporting the conclusions of this article will be made available by the authors, without undue reservation.

## Capsule Summary Statement

Families of organ donors play an important role in the deceased organ donation process. Little is known about the care for donor families. Although there are differences between the countries, families are provided with information about the transplant results and remembrance ceremonies are organised. Aftercare for donor families could improve and be more structural organised. Meetings between donor families and recipients exist. With this inventory of 15 European countries we gain insight in the daily practise, which is important, to learn from other countries and to know where future research should focus on, like the experience and needs of donor families.
