# Corrigendum: An Inventory of Deceased Donor Family Care and Contact Between Donor Families and Recipients in 15 European Countries

**DOI:** 10.3389/ti.2022.10717

**Published:** 2022-08-05

**Authors:** Tineke Wind, Nichon Jansen, Anne Flodén, Bernadette Haase-Kromwijk, David Shaw, Dale Gardiner

**Affiliations:** ^1^ Maastricht University Medical Centre, Maastricht, Netherlands; ^2^ Institute of Health and Care Science, Dutch Transplant Foundation, Leiden, Netherlands; ^3^ Institute of Health and Care Science, University of Gothenburg, Gothenburg, Sweden; ^4^ Department of Anaestesiology, Södra Älvsborgs Hospital, Borås, Sweden; ^5^ Institute of Biomedical Ethics, University of Basel, Basel, Switzerland; ^6^ Department of Health, Ethics and Society, Care and Public Health Research Institute, Maastricht University, Maastricht, Netherlands; ^7^ Nottingham University Hospitals NHS Trust, Nottingham, United Kingdom

**Keywords:** organ donation, donor family care, contact donor and recipient, remembrance ceremonies, family after care

In the original article, the reference for “Delgado J, Molina-Perez A, Shaw D, Rodriguez-Arias D. *The Role of the Family in Deceased Organ Procurement. A Guide for Clinicians and Policy Makers*. Alphen aan den Rijn, Netherlands: Transplantation (2019)” (reference 4) incorrectly included “Alphen aan den Rijn, Netherlands:” and did not include the DOI.

The full reference is “Delgado J, Molina-Perez A, Shaw D, Rodriguez-Arias D. The Role of the Family in Deceased Organ Procurement: A Guide for Clinicians and Policymakers. *Transplantation* (2019) 103(5):e112–18. doi: 10.1097/TP.0000000000002622.”

The authors apologize for this error and state that this does not change the scientific conclusions of the article in any way. The original article has been updated.
